# Extremely low frequency–electromagnetic fields promote chondrogenic differentiation of adipose-derived mesenchymal stem cells through a conventional genetic program

**DOI:** 10.1038/s41598-024-60846-5

**Published:** 2024-05-03

**Authors:** Lucrezia Zerillo, Concetta Claudia Coletta, Jessica Raffaella Madera, Gabriella Grasso, Angelapia Tutela, Pasquale Vito, Romania Stilo, Tiziana Zotti

**Affiliations:** 1 Dipartimento di Scienze e Tecnologie, Università Degli Studi del Sannio, Via dei Mulini, 82100 Benevento, Italy; 2 Genus Biotech, Università Degli Studi del Sannio, Benevento, Italy

**Keywords:** Mesenchymal stem cells (MSCs), Extremely low frequency–electromagnetic fields (ELF–EMFs), Adipose-derived stem cells (ADSCs), Chondrogenesis, Joint disease, Cartilage development, Differentiation, Adult stem cells, Mesenchymal stem cells, Stem-cell differentiation

## Abstract

Progressive cartilage deterioration leads to chronic inflammation and loss of joint function, causing osteoarthritis (OA) and joint disease. Although symptoms vary among individuals, the disease can cause severe pain and permanent disability, and effective therapies are urgently needed. Human Adipose-Derived Stem Cells (ADSCs) may differentiate into chondrocytes and are promising for treating OA. Moreover, recent studies indicate that electromagnetic fields (EMFs) could positively affect the chondrogenic differentiation potential of ADSCs. In this work, we investigated the impact of EMFs with frequencies of 35 Hertz and 58 Hertz, referred to as extremely low frequency-EMFs (ELF–EMFs), on the chondrogenesis of ADSCs, cultured in both monolayer and 3D cell micromasses. ADSC cultures were daily stimulated for 36 min with ELF–EMFs or left unstimulated, and the progression of the differentiation process was evaluated by morphological analysis, extracellular matrix deposition, and gene expression profiling of chondrogenic markers. In both culturing conditions, stimulation with ELF–EMFs did not compromise cell viability but accelerated chondrogenesis by enhancing the secretion and deposition of extracellular matrix components at earlier time points in comparison to unstimulated cells. This study showed that, in an appropriate chondrogenic microenvironment, ELF–EMFs enhance chondrogenic differentiation and may be an important tool for supporting and accelerating the treatment of OA through autologous adipose stem cell therapy.

## Introduction

Cartilage is an avascular and aneural connective tissue distributed across diverse anatomical locations, including synovial joints, the spine, the ribs, the external ear, the nose, and the airways. It provides essential functions such as mechanical support and joint lubrication and contributes to bone formation during developmental processes ^1,2^. Throughout the lifespan, cartilage undergoes physiological remodelling mediated by chondrocytes, which drive the synthesis and turnover of extracellular matrix (ECM) constituents. Nevertheless, cartilage has limited self-renewal potential, and the capacity of chondrocytes to promote tissue remodelling progressively diminishes with age, increasing the risk of gradual degeneration and damage to joint surfaces^3^. The structure and organization of the ECM are fundamental to normal cartilage function.

Pathologies involving cartilaginous tissue frequently result in substantial alterations to ECM architecture, which compromise tissue structure and functions. Cartilage-related disorders can either directly manifest in the presence of genetically inherited mutations of ECM genes, or may arise as a consequence of pathological mechanisms affecting neighbouring tissues, as evidenced in osteochondritis dissecans and inflammatory arthropathies^1^. Furthermore, traumatic joint injuries and age-related manifestations, when neglected, can compromise tissue functionality and lead to joint pain, resulting in the development of a pathological condition commonly known as osteoarthritis (OA)^4^. OA is characterized by the progressive alteration of cartilage-specific-ECM and tissue deterioration, and effective therapies for treating this disease are currently lacking^5,6^.

In addition, during OA pathogenesis, the release of inflammatory cytokines and proteases induces ECM active remodelling processes, which further change cartilage composition. Such alterations exacerbate disease progression by affecting natural chondrogenesis, which is driven by resident mesenchymal stem cells (MSCs) ^7^. Many studies have investigated novel techniques aimed at enhancing tissue repair processes, with a specific focus on the potential benefits of employing stem cells in the treatment of joint diseases^8,9^. The microenvironment governing stem cell differentiation can involve cell–matrix adhesions or cell–cell interactions^10^. A conducive microenvironment plays a crucial role in supporting MSC survival, commitment, and differentiation ^10,11^. Adipose-derived stem cells (ADSCs) show promising differentiation potential toward mesenchymal lineages, such as chondrocytes^12^, thus representing a means of counteracting osteoarticular degeneration. The MSC-based tissue engineering is currently focusing on the generation of functional hyaline cartilage through the commitment of stem cells toward the chondrogenic lineage, to facilitate the subsequent formation of a cartilaginous matrix, mainly formed by Type II Collagen and Glycosaminoglycans (GAGs)^13^. Various chondrogenic induction factors, such as proteins or chemical compounds, have been employed to promote MSC differentiation into the chondrogenic lineage^14^. However, challenges associated with the use of proteins or chemicals include protein denaturation, the potential transportation of pathogens, and the occurrence of undesired side effects^14^. In addition, alternative methods are based on the biophysical stimulation to induce MSC differentiation into specific lineages^14–16^.

Electromagnetic fields (EMFs) may affect the electrical and physical properties of tissues and cells in multiple ways^17–19^. Depending on flux density and intensity, EMFs can be distinguished in static and time-varying/pulsed. Pulsed electromagnetic field (PEMF) stimulation systems have gained approval from the Food and Drug Administration (FDA) for treating bone fractures, particularly nonunion fractures^20^. The ability of EMFs to stimulate bone repair is well known and widely exploited in hospitals and rehabilitation centres worldwide, although the biological mechanism of action is still elusive. Further parameters, such as the frequency range of employed stimuli, as well as their amplitude, modulation, and waveforms, may also influence the interaction between EMFs and biological systems^21^. Based on frequencies, non-ionizing EMF signals can be classified as Extremely Low Frequency fields (ELF, between 1 Hz up and 100 kHz), Radio Frequency fields (RF, 100 kHz–3 GHz), and Microwaves (MW, above 3 GHz)^22^. Recent studies suggest that EMFs modify adult stem cell behaviour^23^ and, more specifically, may positively influence the chondrogenic differentiation of MSCs^24–28^, but the efficacy of ELF–EMFs needs further investigation.

Here, we explore the therapeutic potential of ELF–EMFs in inducing chondrogenic differentiation of ADSCs in two different culture systems: as cell monolayer and as three-dimensional (3D) micromasses, which are an ideal model to investigate chondrogenesis in vitro^29^. Previous literature has demonstrated that the differentiation of MSCs to chondrocytes and the production of cartilage-specific Type II Collagen is highly promoted in micromasses with respect to other 3D culture systems, such as pellets^30^.

The aim of this study is to evaluate whether ELF–EMFs may be a tool to improve the regenerative capacity of MSCs, with important implications in the autologous ADSC therapy, currently used to treat knee OA^31^.

## Results

### ELF–EMFs do not compromise cell viability and positively modulate ECM secretion in ADSC monolayer cultures

**Figure 1:**
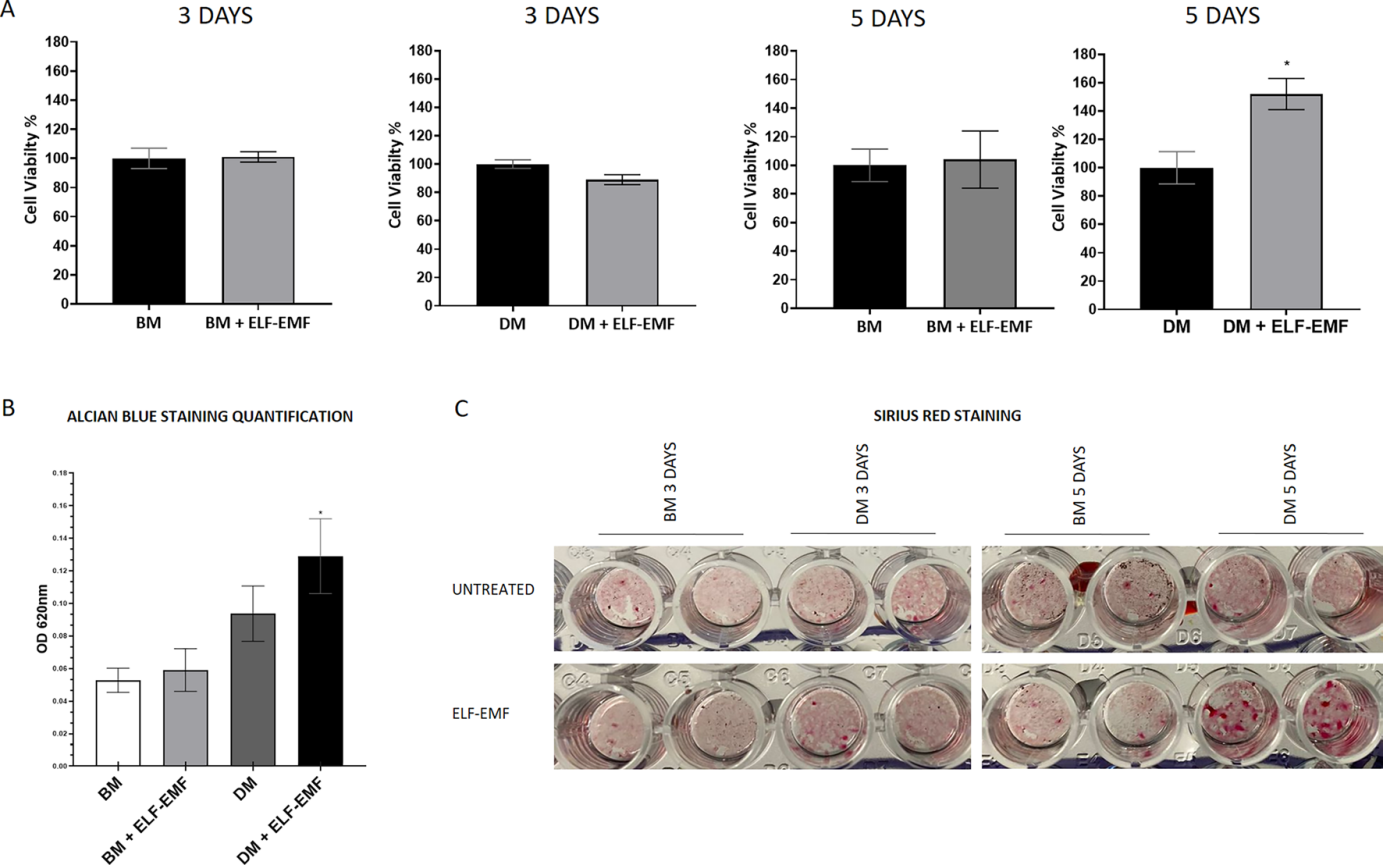
Effect of ELF–EMFs on cell viability and ECM deposition in ADSC monolayer cultures. (**A**) Effect of ELF–EMFs on ADSC viability. Cells cultured in either BM or DM were treated daily with ELF–EMFs for 3 or 5 days. The data are presented as the mean ± standard deviation (SD). The experiments were carried out in triplicate. Statistical significance was determined by parametric unpaired t-test *P value = 0.0047. (**B**) Quantification of GAGs secreted by ADSCs through alcian blue staining. ADSCs cultured in either BM or DM were treated daily with ELF–EMFs for 3 days. After staining with alcian blue, the absorbance at 620 nm was measured to quantify GAG deposition. The data are presented as the mean ± SD. The experiments were performed in triplicate. Statistical significance was determined by one-way ANOVA followed by Tukey’s multiple comparison tests. DM + ELF–EMFs versus. BM *P value = 0.0308. (**C**) Analysis of Collagen deposition by Sirius red staining of ADSCs. Representative image of Sirius red-stained ADSCs cultured in either BM or DM, stimulated or not with ELF–EMFs each day. Analyses were performed 3 or 5 days after the beginning of treatment.

To investigate the impact of ELF–EMF stimulation on the viability of ADSC monolayers, we performed MTT assays following daily 36 min ELF–EMF treatment for a total duration of 3 or 5 days. ADSCs were cultured in either Basal Medium (BM) or Differentiation medium (DM), which induces chondrogenesis. As shown in Fig. 1A, cell viability was not affected by daily ELF–EMF treatment after 3 days in ADSCs cultured in both BM and DM. In contrast, after 5 days, ADSCs cultured in DM and treated with daily ELF–EMFs showed significantly greater viability (*P* value = 0.0047). Then, we monitored ADSC differentiation into chondrocytes by quantifying the deposition of GAGs in the ECM through alcian blue staining. ADSCs in either BM or DM were treated every day for 3 days with ELF–EMFs and stained for GAGs. In comparison to ADSCs cultured in BM, we observed higher GAG secretion in ADSCs cultured in DM-cultured cells, which is further significantly increased by ELF–EMF stimulation (*P* value = 0.0308; Fig. 1B).

Subsequently, we also performed a Sirius red picrate colorimetric assay to evaluate collagen deposition in the ECM by ADSCs subjected to daily ELF–EMF treatment after 3 or 5 days (Fig. 1C). Visual inspection revealed distinct colorimetric differences among the experimental conditions, with higher intensity of red staining in ADSCs cultured in DM + ELF–EMFs after 3 and 5 days. Taken together, these findings suggest that ELF–EMF treatment enhances ECM secretion in ADSCs cultured in DM.

### ELF–EMFs affect gene expression of chondrogenic-related markers *RUNX2* and *ACAN* and induce an increase in COL2A1 protein levels

**Figure 2:**
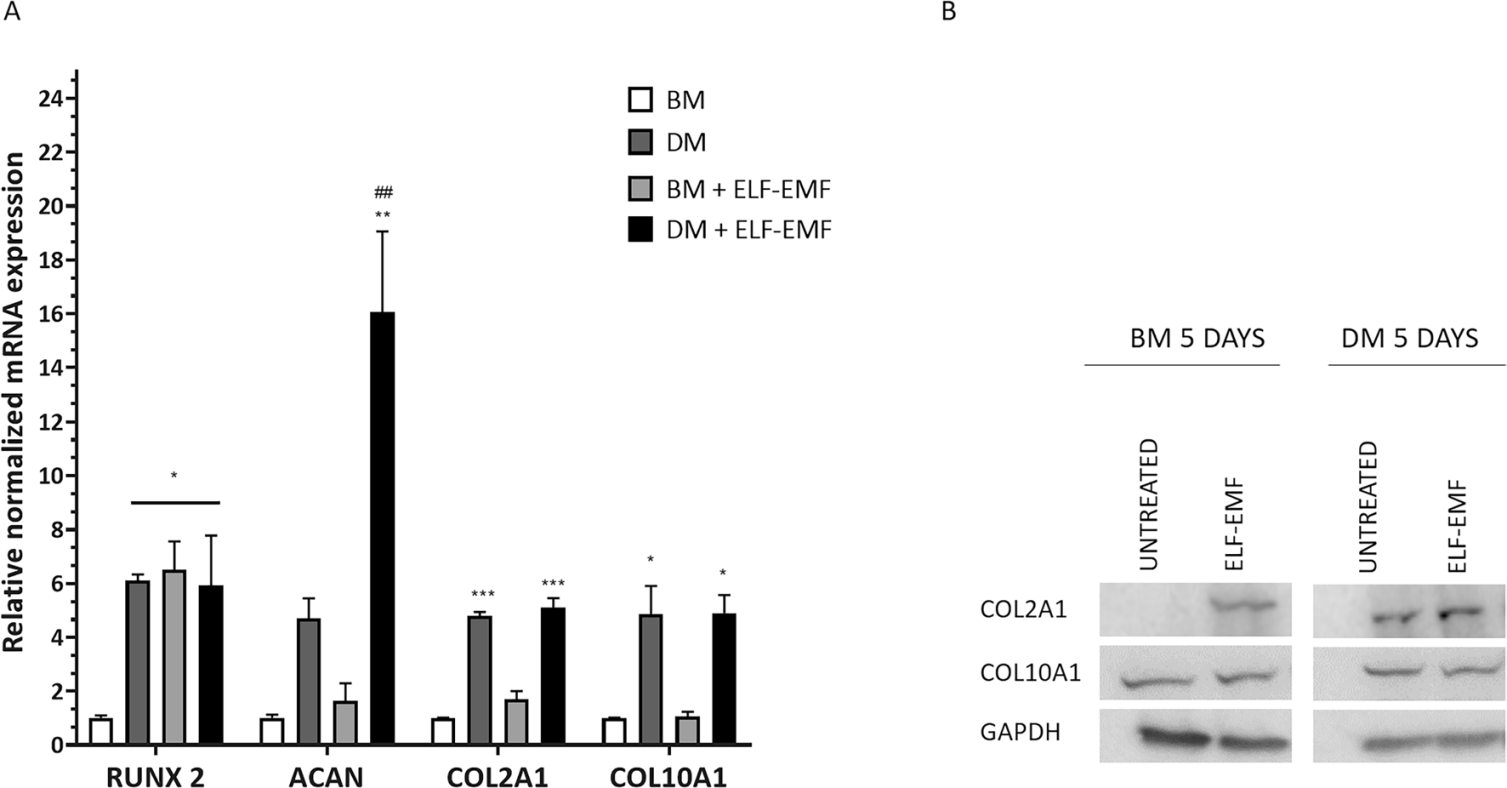
Effect of ELF–EMFs on chondrogenic marker expression in ADSC monolayer cultures. (**A**) Gene expression analysis of *RUNX2, ACAN, COL2A1,* and *COL10A1* in ADSCs cultured in either BM or DM with or without daily treatment with ELF–EMFs for 5 days. The data are presented as the mean ± SD. The experiments were performed in triplicate. Statistical significance was determined by one-way ANOVA followed by Tukey’s multiple comparison tests. For *RUNX2*: DM, BM + ELF- EMFs, and DM + ELF–EMFs versus BM *P value < 0.05. For *ACAN*: DM + ELF–EMFs versus BM **P value < 0.01 and DM + ELF–EMFs versus DM ^##^ *P* value < 0.01. For *COL2A1*: DM, and DM + ELF–EMFs versus BM ***P value < 0.005. For *COL10A1*: DM, and DM + ELF–EMFs versus BM *P value < 0.05. (**B**) Representative image of COL2A1 and COL10A1 protein expression in ADSCs cultured in either BM or DM and exposed to daily treatment with ELF–EMFs for 5 days. The expression level of GAPDH was used as a loading control. The experiments were performed in triplicate. The full-length blots corresponding to (**B**) are presented in Supplementary Fig. S1.

The impact of ELF–EMFs on ADSCs differentiation was also investigated by analysing the gene expression levels of the chondrogenic markers *RUNX2*, *ACAN*, *COL2A1,* and *COL10A1* through RT-qPCR (Fig. 2A). All the markers were significantly up-regulated in ADSCs cultured in DM compared with gene expression levels observed in BM-cultured cells, whereas the treatment with ELF–EMFs affects the mRNA expression levels of *RUNX2* and *ACAN* in ADSCs cultured in BM and DM, respectively. In contrast, ELF–EMFs do not modify *COL2A1* and *COL10A1* expression (Fig. 2A).

As collagen deposition is not only due to gene upregulation^32–35^, we assessed the protein levels of COL2A1 and COL10A1 through immunoblotting (Fig. 2B). As expected, ADSCs in DM had higher levels of COL2A1 than cells cultured under basal conditions. Interestingly, daily stimulation with ELF–EMFs for 5 days was associated with the accumulation of COL2A1 in ADSCs cultured in both BM and DM (Fig. 2B). Conversely, the expression level of COL10A1 was roughly invariant under all the experimental conditions (Fig. 2B).

### ELF–EMFs actively shape chondrogenic differentiation without compromising cell viability in 3D-cultured ADSCs

**Figure 3:**
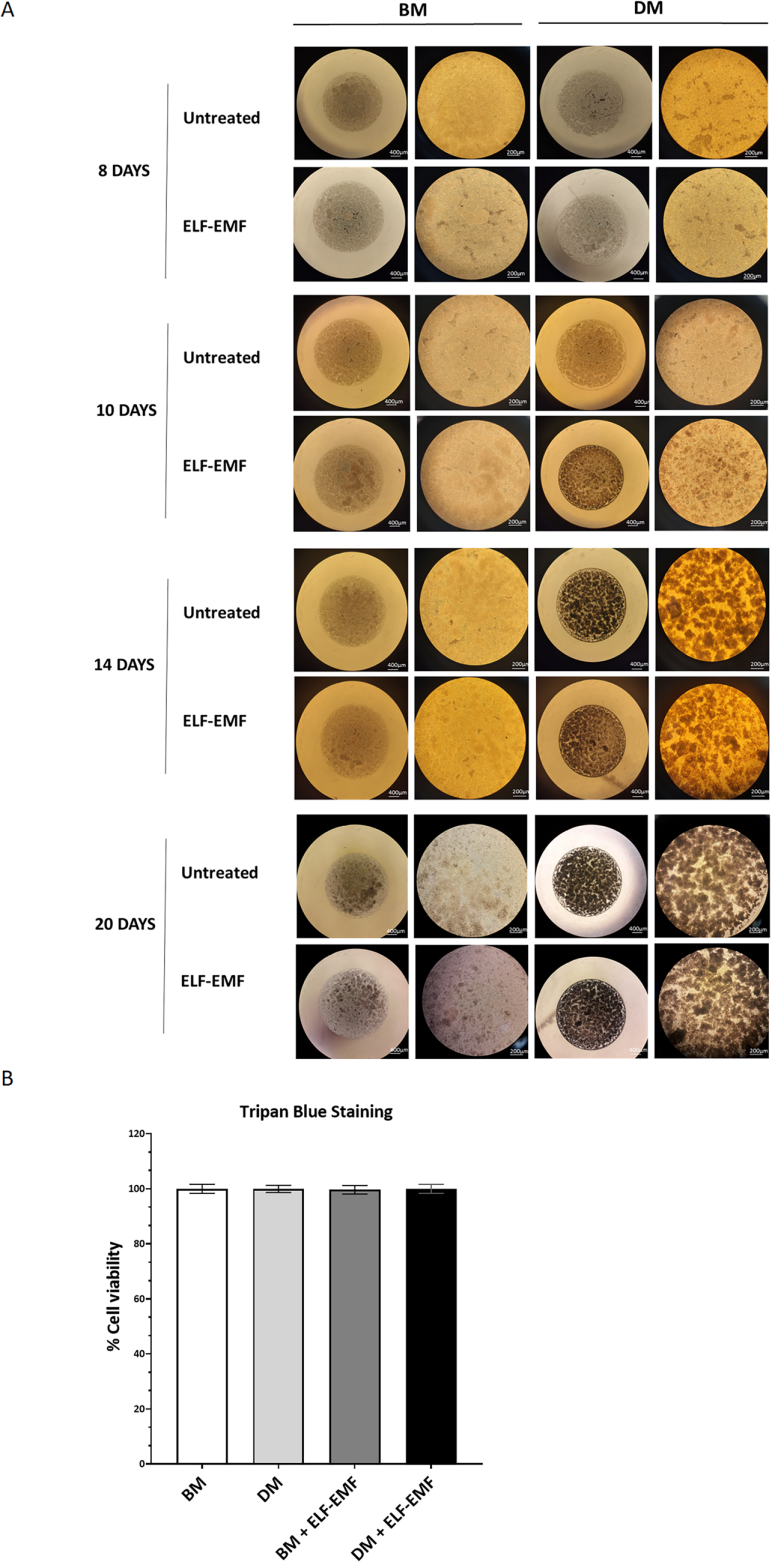
Effect of ELF–EMFs on morphology and viability of ADSCs cultured as 3D micromasses. (**A**) Representative micrographs illustrating chondrogenic differentiation in ADSCs cultured in 3D micromasses maintained in either BM or DM and subjected to ELF–EMFs each day for 21 days. Morphological evaluations were conducted using phase-contrast optical microscopy 8, 10, 14, and 20 days after the initiation of ELF–EMF treatment. Micromasses were examined at 4 × and 10 × magnifications. (**B**) Analysis through Trypan blue staining of 3D cultures of ADSCs subjected to daily treatment with ELF–EMF for 21 days. The analysis illustrates the percentage of viable cells under various experimental conditions. The data are presented as the mean ± SD. The experiments were carried out in triplicate.

We also analysed the effect of ELF–EMFs on the differentiation of ADSCs cultivated as 3D micromasses, an experimental model that closely mimics in *vivo* mechanisms^29,30^. ADSC micromasses were cultured in either BM or DM, with or without daily stimulation with ELF–EMFs. The morphology of the micromasses was assessed through phase-contrast optical microscopy 8, 10, 14, and 20 days after the initiation of stimulation (Fig. 3A). Up to day 8, no significant differences in the morphology of the cell masses were observed among the described experimental conditions. On day 10, we noticed the accumulation of brownish granules in the ADSC micromasses cultured in DM and treated with ELF–EMFs, suggesting that the cells initiate the production and deposition of ECM components (Fig. 3A). From day 14 onwards, all micromasses in DM exhibited dark spots, with no further morphological differences observed among the various experimental conditions until the end of treatment (Fig. 3A, lower panels). Subsequently, we analysed the cell viability of 3D cultures of ADSCs maintained in BM or DM and subjected to daily ELF–EMF treatment for 21 days using trypan blue staining (Fig. 3B). In our experimental conditions, stimulation with ELF–EMFs did not compromise the cell viability of micromasses.

### Treatment of 3D-cultured ADSCs with ELF–EMFs accelerates ECM secretion

**Figure 4:**
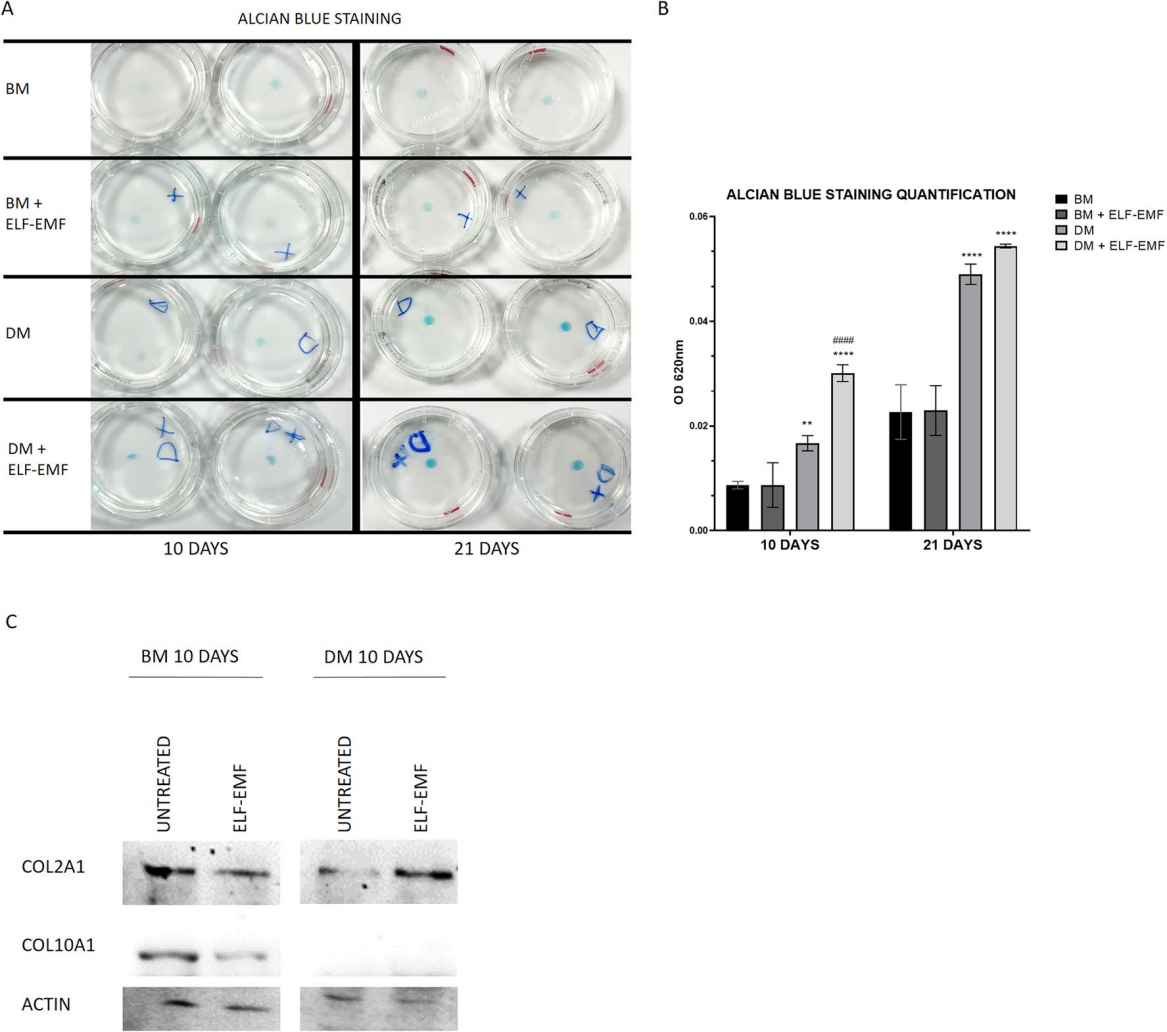
Effect of ELF–EMFs on morphology and ECM deposition of ADSCs cultured as 3D micromasses. (**A**) Representative image of alcian blue staining of ADSC micromasses maintained in either BM or DM, and stimulated or not with ELF–EMFs for 10 and 21 days. (**B**) Quantification of GAGs from alcian blue staining of ADSC micromasses as in (**A**). The data are presented as the mean ± SD. The experiments were performed in triplicate. Statistical significance was determined by one-way ANOVA followed by Tukey’s multiple comparison test. For staining at 10 days: DM versus BM **P value = 0.0026; DM + ELF–EMFs versus BM ****P value < 0.0001; DM + ELF–EMFs versus DM ^####^*P* value < 0.0001. For staining at 21 days: DM, and DM + ELF–EMFs versus BM ****P value < 0.0001. (**C**) COL2A1 and COL10A1 protein levels in 3D-cultured ADSCs maintained in either BM or DM and stimulated or not with ELF–EMFs for 10 days. Actin levels were used for normalization. The full-length blots corresponding to Fig. 4C are presented in Supplementary Fig. S2.

Next, we evaluated the effect of ELF–EMFs on the chondrogenesis of ADSC micromasses. Since previous experiments have shown that micromasses in DM + ELF–EMFs start the production of ECM-like granules 10 days after the beginning of treatment and culminate in ECM deposition on day 21, we performed alcian blue staining at the same time points. As shown in Fig. 4A, micromasses cultured in DM had a stronger blue color intensity than those grown in BM, indicating higher GAG deposition and differentiation. Furthermore, micromasses in DM + ELF–EMFs appear even more intensely stained with respect to all other conditions. The quantification of the alcian blue staining by measuring absorbance at 620 nm confirmed this evidence (Fig. 4B). On day 10, the quantitative analysis revealed a significant increase in GAG deposition in the micromasses cultured in both DM and DM + ELF–EMFs with respect to micromasses in basal conditions (*P* value = 0.0026, and *P* value < 0.0001, respectively). Interestingly, ELF–EMF treatment significantly enhances GAG secretion in micromasses cultured in DM (*P* value < 0.0001). On day 21, we noticed a significant increase in GAG staining in all micromasses maintained in DM (both stimulated or not with ELF–EMFs) when compared to BM-culturing conditions (*P* values < 0.0001), but no considerable differences were observed between DM and DM + ELF–EMFs. At both time points, we could not detect any differences in alcian blue staining of micromasses cultured in BM + ELF–EMFs with respect to those in BM (Fig. 4B).

Subsequently, the protein levels of COL2A1 and COL10A1 were analyzed in ADSC micromasses cultured in either BM or DM, and stimulated or not with ELF–EMFs for 10 days (Fig. 4C). The stimulation with ELF–EMFs of micromasses cultured in BM induces no significant changes in COL2A1 levels and a slight reduction of COL10A1. Differently, in differentiation conditions COL2A1 protein accumulates after stimulation with ELF–EMFs, whereas COL10A1 protein expression was almost undetectable (Fig. 4C).

### ELF–EMF exposure enhances *IL6* expression following TLR4 activation in ADSCs

**Figure 5:**
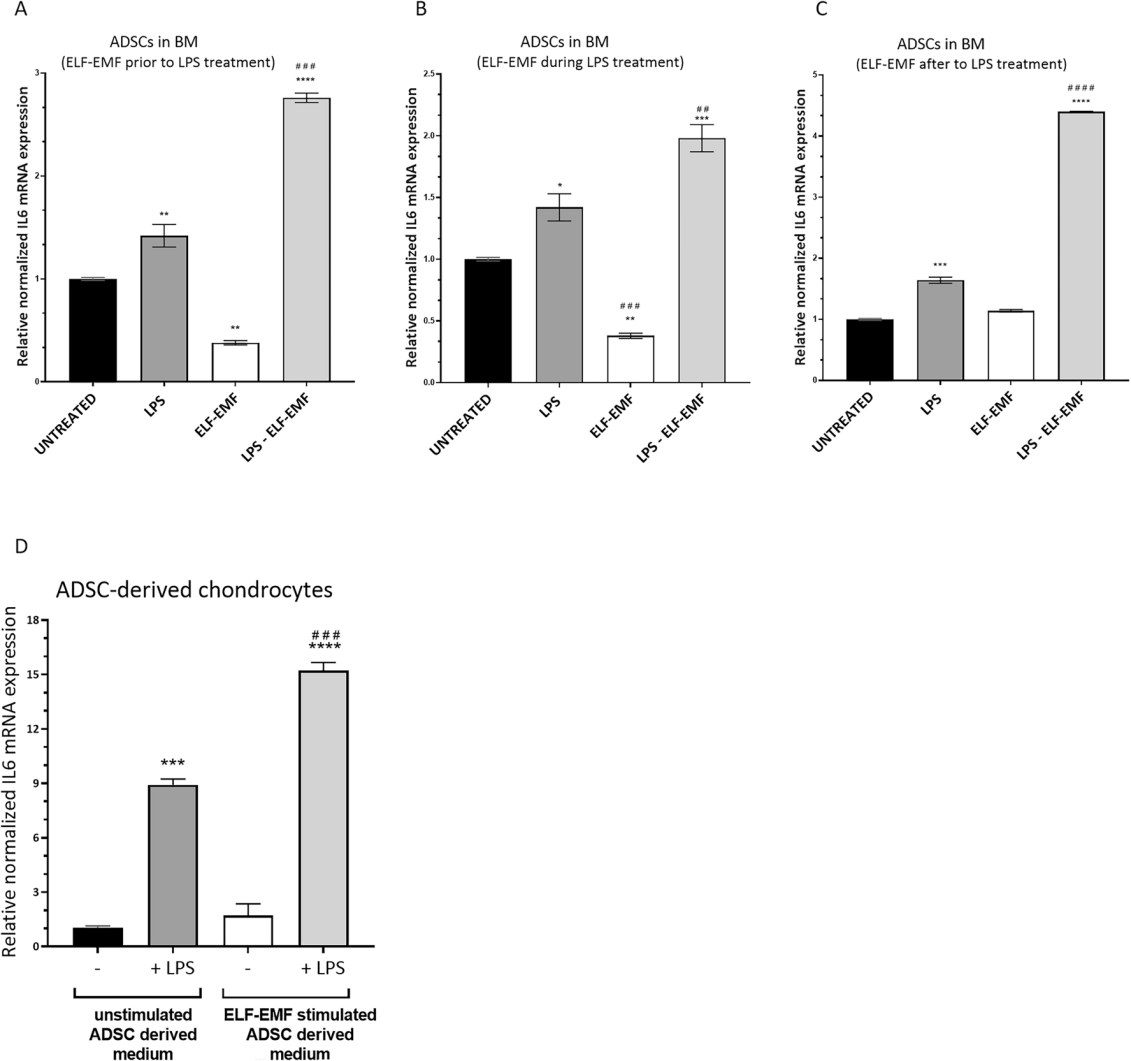
Effect of ELF–EMFs on *IL6* expression in ADSCs. (**A**–**C**) Gene expression analysis of *IL6* in ADSCs cultured in BM, treated with or without 5 µg/mL LPS and stimulated with ELF–EMFELF–EMFs. In (**A**), ADSCs were once stimulated or not for 36 min with ELF–EMFs, and, right after, LPS was added or not to BM for 5 h. In (**B**) LPS was added or not to BM and left for 5 h. ELF–EMF stimulation was used, or not, once during treatment for 36 min, 1 h after LPS addition. In (**C**), ADSCs were first treated or not with LPS for 5 h, and, right after, cells were once stimulated or not with ELF–EMFs for 36 min. For (**A**) and (**B**) mRNA extraction was performed right after the end of LPS stimulation. For (**C**) mRNA extraction was performed 12 h after the end of LPS stimulation. The data are presented as the mean ± SD (*n* = 3). Statistical significance was determined by one-way ANOVA followed by Tukey’s multiple comparison test. For (**A**): LPS, and ELF–EMFs versus Untreated **P value < 0.01; LPS + ELF–EMFs versus LPS ^###^*P* value < 0.001. For (**B**): LPS versus Untreated *P value = 0.0202; ELF–EMFs versus Untreated **P value = 0.0049; LPS + ELF–EMFs versus Untreated ***P value = 0.0008; LPS + ELF–EMFs versus LPS ^##^*P* value = 0.0072. For (C): LPS versus Untreated ***P value = 0.0001; LPS + ELF–EMFs versus Untreated ****P value < 0.0001; LPS + ELF–EMFs versus LPS ^####^*P* value < 0.0001. (**D**) Gene expression analysis of *IL6* in ADSC-derived chondrocytes treated with LPS in ADSC-derived medium. ADSCs were differentiated into chondrocytes for 14 days in DM. Then, they were stimulated for 6 h with a preconditioned medium with or without 5 µg/mL LPS. The preconditioned medium was prepared as follows: undifferentiated ADSCs were cultured in BM for 24 h and stimulated or not with ELF–EMFs once. The data are presented as the mean ± SD (*n* = 3). Statistical significance was determined by one-way ANOVA followed by Tukey’s multiple comparison test. Unstimulated ADSC-derived medium + LPS versus Unstimulated ADSC-derived medium ***P value = 0.0002; ELF–EMF stimulated ADSC-derived medium + LPS versus Unstimulated ADSC-derived medium ****P value < 0.0001; ELF–EMF stimulated ADSC-derived medium + LPS versus Unstimulated ADSC-derived medium + LPS ^####^*P* value < 0.0001.

As TLR4 and IL6 have been reported to play key roles in chondrogenesis^36^, we evaluated the effect of ELF–EMFs on the induction of *IL6* gene expression after TLR4 activation. In brief, ADSCs were treated with 5 µg/mL Lipopolysaccharide (LPS, a TLR4 agonist) for 5 h and stimulated with ELF–EMFs before, during, or after LPS treatment. The transcriptional level of the *IL6* gene was measured through RT-qPCR. As expected, LPS induced *IL6*, whereas stimulation with ELF–EMFs alone slightly reduces (Fig. 5A,B) or has no effects (Fig. 5C) on *IL6* mRNA. More interestingly, when ELF–EMFs are used in combination with LPS treatment, we observed a statistically significant increase in *IL6* gene expression with respect to exposure to LPS alone under all the experimental conditions (Fig. 5A–C).

Recent work has demonstrated that ADSCs can release various biologically active molecules, influencing the cell milieu through a paracrine mechanism^37^. Consequently, we evaluated the impact of ADSC-derived medium on the *IL6* expression levels in chondrocytes. To this purpose, ADSCs were differentiated into chondrocytes for 14 days in DM. Then, they were stimulated for 6 h with a preconditioned medium combined or not with 5 µg/mL LPS. Such preconditioned medium, referred to as ADSC-derived medium, was prepared from undifferentiated ADSCs cultured in BM for 24 h and stimulated or not with ELF–EMFs once. Interestingly, the medium derived from undifferentiated ADSCs that were treated with ELF–EMFs significantly enhances *IL6* expression induced by exposure to LPS in chondrocytes (Fig. 5D).

## Discussion

Cartilage is a connective tissue crucial for maintaining musculoskeletal health. Degeneration of cartilage tissue is a hallmark of widespread global conditions such as OA. Due to its avascular and aneural nature, cartilage-related pathologies are challenging to diagnose and difficult to treat either surgically or pharmacologically ^1^. The limited self-renewal potential of articular hyaline cartilage poses a significant challenge for orthopaedic research ^38^. Recent studies have explored new techniques to enhance tissue repair processes, with a focus on the potential advantages of using stem cells in joint disease treatment. ADSCs are a promising cellular source for cartilage regeneration due to their accessibility ^39–41^.

EMFs are known to significantly impact the chondrogenic differentiation of ADSCs ^27,42^, but it is not entirely clear whether ELF–EMFs have the same effects. Therefore, we investigated the role of ELF–EMFs in promoting the chondrogenesis of ADSCs in vitro. After an initial study on monolayer-cultured ADSCs, we extended our investigation to ADSCs cultured as 3D micromasses which are an ideal model for in vitro chondrogenic studies^29,30^.

The experiments carried out in the two culturing conditions had different durations for two main reasons. Firstly, the cultivation of ADSCs in DM as monolayer cannot last longer than 7–10 days, as after this time in DM, cells naturally tend to aggregate and form spheroids^29^. Therefore, to minimize the differences related to the inhomogeneity of the biological system treated with ELF–EMFs, we performed the experiments in monolayer within 5 days. Secondly, the induction of differentiation in the monolayer for shorter periods allows us to investigate some of the initial events of the differentiation process, including the up-regulation in the expression levels of factors associated with chondrogenic differentiation. Conversely, we extended the treatment time to 10 and 21 days for micromass analysis, so that we could monitor ECM deposition during the subsequent stages of chondrogenesis and when cells are terminally differentiated into chondrocytes. Our findings suggest that ELF–EMF daily treatment of ADSCs in monolayer and 3D micromasses does not compromise cell viability, consistent with previous literature^42^. In particular, we found that ELF–EMFs induce a significant increase in cell viability on ADSCs cultured in DM as a monolayer, which can partially explain the subsequent increase of GAG and collagen deposition, as assessed by alcian blue and Sirius red staining, respectively.

The increase of GAG secretion was also observed on days 10 and 21 in ADSC micromasses cultured in DM and stimulated with ELF–EMFs, without significant changes in the number of viable cells, indicating that ELF–EMFs would rather accelerate ECM deposition in the micromass model. On the other hand, after the complete differentiation of ADSCs into chondrocytes, no marked changes in morphology and GAG deposition are observed on either ELF–EMF stimulated or unstimulated micromasses.

Taken together, these results suggest that chondrogenic differentiation occurs earlier in ELF–EMF-treated cells than in unstimulated cells, as also proposed by Iorio et al.^42^. Nevertheless, in contrast with them^42^, the analysis of chondrogenic marker gene expression (*COL2A1, ACAN, RUNX2,* and *COL10A1*) showed significant variations only in *RUNX2* and *ACAN* genes, whose expression is up-regulated following exposure to ELF–EMFs in cells cultured in BM and DM, respectively. Together with the data showing increased protein levels of COL2A1 in cells treated with DM + ELF–EMFs, gene expression analyses indicate that the effect of ELF–EMFs on the secretion of ECM components occurs through both transcriptional and post-transcriptional mechanisms. On the one hand, ELF–EMFs specifically enhance proteoglycan synthesis through the induction of *ACAN* gene expression and, on the other hand, ELF–EMFs may influence COL2A1 protein maturation, stability, and extracellular deposition.

The effect of ELF–EMFs on *RUNX2* gene expression in ADSCs cultured in BM is particularly relevant, as this transcription factor is essential for both cartilage and bone formation. In particular, *RUNX2* deficiency is associated with the absence of osteoblast differentiation^43,44^ and delayed chondrogenesis^45,46^. The induction of the *RUNX2* gene is essential for chondrocyte maturation and precedes the induction of *COL10A1* in hypertrophic chondrocytes, which transdifferentiate into osteoblasts^47–49^. In our experimental setting, we observed an increase in *RUNX2* mRNA that was not associated with either concomitant activation of the *COL10A1* gene or stabilization of its protein product in undifferentiated mesenchymal cells, suggesting that chondrogenesis is potentially initiated, or at least facilitated, by ELF–EMFs but that chondrogenesis does not proceed spontaneously toward endochondral ossification steps. Indeed, in micromasses maintained in DM, the protein levels of COL10A1, which is typically overexpressed in hypertrophic conditions ^45,50^, are almost undetectable, confirming that ELF–EMF stimulation accelerates chondrogenesis but does not induce hypertrophy.

Therefore, it is conceivable that the acceleration of chondrogenesis induced by ELF–EMFs occurs through the induction of a conventional genetic program of differentiation and not through further non-canonical genetic and/or epigenetic mechanisms. However, the involvement of ELF–EMF-induced *RUNX2* up-regulation in MSC differentiation is fascinating and requires further and more detailed investigation.

Moreover, the increase in LPS-induced *IL6* expression observed in ADSCs under ELF–EMF stimulation might have important implications. In addition to its role in inflammation, IL6 has recently been shown to promote the differentiation of human MSCs into chondrocytes^51^. High levels of IL6 in proximity to MSC-like cells within cartilage contribute to tissue homeostasis and self-repair, promoting chondrogenic differentiation^52^. Intriguingly, we observed an up-regulation of *IL6* both in ADSCs exposed to ELF–EMFs in combination with LPS and in differentiated chondrocytes treated with LPS in ELF–EMF-stimulated ADSC-derived medium. This evidence points to an additional mechanism by which chondrogenesis might be affected by ELF–EMFs. However, the molecular details of this relationship, if any, require further analysis.

A main limitation of the present study is that the gene expression of chondrogenic and hypertrophy markers was not analysed in micromasses due to the small amount of RNA extracted. Therefore, we could not confirm the effect of ELF–EMFs on the transcriptional program activated in micromasses during chondrogenesis.

However, exploring new interventions aimed at enhancing MSC performance, both in terms of repair and regeneration capacity and by modulating immune and inflammatory responses, will significantly contribute to regenerative medicine and tissue repair approaches. Our results indicate that, in a suitable chondrogenic microenvironment, physical stimuli such as ELF–EMFs can be used as a tool to enhance the chondrogenic differentiation of ADSCs. Future studies are needed to evaluate in which way different treatment conditions (duration, number of pulses, frequencies, and waveforms) may modify and assist the molecular processes associated with chondrogenesis.

## Methods

### Cell cultures and reagents

ADSCs sourced from StemPro^®^ Human Adipose-Derived Stem Cells (Lot No. 1001005; Invitrogen) were cultured in MesenPRO RS™ Basal Medium (BM). When specified, ADSCs were subjected to chondrogenic differentiation using the StemPro^®^ Chondrogenesis Differentiation Kit, referred to as Differentiation Medium (DM). According to the manufacturer’s instructions, this standardized process encompasses both expansion and differentiation phases into matrix-forming chondrocytes.

For *IL6* gene expression analysis, ADSCs were treated for 5 h with 5 µg/mL Lipopolysaccharide from Escherichia coli 0111:B4 purchased by SIGMA-ALDRICH (Saint-Louis, MO, USA).

### Monolayer cultures

ADSCs were cultured in BM supplemented with glutamine and growth factors (MesenPRO RS™ Growth Supplement). Following cell adhesion, whenever described in the experimental conditions, the cell medium was replaced with StemPro^®^ Chondrogenesis DM. The induction of differentiation with DM has been carried out for either 3 or 5 days, as indicated. Monolayer cultures in 12-well plates were used for subsequent gene and protein expression analyses (SDS‒PAGE and RT‒qPCR), while 96-well plates were used for colorimetric analyses of ECM components (Alcian blue staining and Sirius red staining) and cell viability assays (MTT assay).

### 3D cultures

3D ADSC micromasses were prepared as in Zhang et al.^30^. Briefly, monolayer-cultured cells were detached, and suspended in BM at a density of 2 × 10^7^ cells/mL. To create micromasses, 12.5 μL droplets were placed in 60 mm-plates and let them adhere for 2 h in BM. Depending on experimental conditions, BM or DM was added after adhesion and changed every 3 or 4 days. 3D micromasses were then stimulated daily for 36 min with ELF–EMFs and micro-photographed on days 8, 10, 14, and 20 with 4 × and 10 × magnification. The induction of differentiation with DM has been carried out for either 10 or 21 days, as indicated. According to the manufacturer’s instructions, complete differentiation of ADSCs into chondrocytes is reached after 20 days in DM.

### ELF–EMF exposure system

For UL-EMF stimulation, ADSCs cultured in plastic TC plates were daily stimulated for 36 min with an electro-medical device that combines static and alternating sinusoidal EMFs, thereby generating an Ion Cyclotron Resonance-Like effect. In our experimental system, the cyclotron resonance has been determined for Calcium and Magnesium ions according to the following formula:

$$2\pi {f}_{c}=\frac{q}{m}\times B$$

where ${f}_{c}$ is the resonance frequency (Hz), $q$ is the charge on the ion (C), $m$ is the mass of the ion (Kg), and $B$ is the static magnetic Field (T, μT or Gauss). Therefore, the device was set up to create pulsed UL-EMFs (with 100 μT intensity and 35 Hz and 58 Hz frequencies) in the presence of a static EMF (with 46μT intensity). The sinusoidal shape of burst signals was chosen as per previous publications^53–57^. Stimulation was performed for 5 days when ADSCs were cultured as monolayer, and for 21 days when cells were cultured as 3D micromasses.

### Cellular morphology analysis

The impact of treatment on cellular morphology was monitored daily through an optical phase-contrast microscope, with untreated cultures serving as controls. Morphological evaluations were conducted at 4 × or 10 × magnification.

### Cell viability assay

Cell viability in monolayer cultures was assessed utilizing the Invitrogen™ CyQUANT™ MTT Proliferation Assay Kit following the manufacturer’s instructions.

The Trypan blue exclusion assay was used to assess the viability of the cells in 3D culture. Viable and dead cells stained with Trypan blue were quantified using a Burker chamber. Experiments were performed in triplicate, and the data are presented as mean ± SD.

### Gene expression analysis

Gene expression analysis was performed as previously described^58^. Briefly, total RNA was isolated from ADSCs using TRIzol (Invitrogen, Carlsbad, CA, USA). Reverse transcription was performed on 1 μg of RNA with OneScript^®^ Plus cDNA Synthesis Kit (ABM, Richmond, BC, Canada). Then, 30 nanograms of cDNA were used in the subsequent amplification step along with 300 nM of each primer in a total volume of 10 μl. The sequences of primers used are listed in Table 1.

**Table 1:** Sequences of primers used in the present study.

Gene	Forward 5′-3′	Reverse 5′-3′
RUX2	GGT CAG ATG CAG GCG GCC	TAC GTG TGG TAG CGC GCT
GAPDH	ACC CAG AAG ACT GTG GAT GG	TTC TAG ACG GCA GGT CAG GT
ACAN	TAC ACT GGC GAG CAC TGT AAC	CAG TGG CCC TGG TAC TTG TT
COL2A1	GTG AAC CTG GTG TCT CTG GTC	TTT CCA GGT TTT CCA GCT TC
COL10A1	CAC CTT CTG CAC TGC TCA TC	GGC AGC ATA TTC TCA GAT GGA
IL6	TCAATGAGGAGACTTGCCTG	TGGGTCAGGGGTGGTTATTG

Quantitative PCR was performed using BlastTaq™2X qPCR MasterMix (ABM, Richmond, BC, Canada) in a Real-Time PCR QuantStudio5 (Applied Biosystems, Thermo Fisher Corporation, Waltham, Massachusetts, USA) with the following cycling conditions: 3 min at 95 °C; 40 cycles of 1 s at 95 °C and 10 s at 60 °C (with Plate Read); and a melting curve analysis to confirm specific amplification. The expression levels of the *GAPDH* gene were chosen as a reference, and the relative transcription level of each gene was calculated by using the ΔΔC_T_ method ^59^.

### Immunoblotting

Cells were lysed by using NP-40 lysis buffer (NaCl 120 mM, Tris 20 mM pH 7.5; NP-40 2%) containing protease inhibitors, and insoluble fractions were pelleted by centrifugation at 13,000 rpm for 30 min at 4 °C. Equal amounts of protein extracts were separated via SDS-PAGE (sodium dodecyl sulphate–polyacrylamide gel electrophoresis) and transferred onto nitrocellulose membranes. After blocking, the membranes were incubated overnight at 4 °C with the following specific primary antibodies: anti-COL2A1 (# A1560, ABclonal Technology) and anti-COL10A1 (# A18604, ABclonal Technology). Subsequently, horseradish peroxidase‐conjugated secondary antibodies were used to visualize the protein signals via chemiluminescence. The GAPDH (#VMA0004, Bio-Rad) protein expression levels were used for normalization.

### GAG staining

The cell cultures were washed with PBS before fixation using a PFA 4% solution for 20 min. Subsequently, the cells were incubated for 30 min with 1% Alcian blue in 3% acetic acid. Excess dye was removed using 3% acetic acid and deionized water. Subsequently, the cells were treated with a 3% sodium dodecyl sulfate (SDS) solution followed by homogenization using a shaker at 200 × g for 30 min. The absorbance was read at 620 nm using a microplate reader. Experiments were performed in triplicate, and the data are presented as mean ± SDs.

### Collagen staining

Collagen staining was performed using Sirius red picrate staining (Bio Optica Sirius Red for Collagen and Bile Pigments Kit) following the manufacturer's instructions.

### Statistical analysis

All statistics were performed with GraphPad Prism 8.0. T-test was carried out on data from the MTT assay on monolayer cultures. One-way ANOVA followed by Tukey’s multiple comparison tests was performed to assess significant differences where indicated as in figure legends. A *P* value < 0.05 was considered significant.

### Supplementary Information

Supplementary Figures.

## Data Availability

Data is provided within the manuscript or supplementary information files.

## Abbreviations

ELF–EMFs: Extremely low frequency–electromagnetic fields
ADSCs: Adipose-derived stem cells
MSCs: Mesenchymal stem cells
ECM: Extracellular matrix
BM: Basal medium
DM: Differentiation medium
GAGs: Glycosaminoglycans
COL2A1: Collagen 2A
COL10A1: Collagen 10A1
ACAN: Aggrecan
RUNX2: Runt-related transcription factor 2
