# Supplementary material for: Extremely low frequency–electromagnetic fields promote chondrogenic differentiation of adipose-derived mesenchymal stem cells through a conventional genetic program
Source: Sci Rep. 2024 May 3;14:10182. doi: 10.1038/s41598-024-60846-5 (PMC11068729; doi:10.1038/s41598-024-60846-5)
Supplement: Supplementary file 1 — Supplementary Figures. [file 41598_2024_60846_MOESM1_ESM.pdf]

SUPPLEMENTARY MATERIALS FOR

Extremely low frequency–electromagnetic fields promote chondrogenic differentiation of Adipose-derived Mesenchymal Stem Cells through a conventional genetic program

Lucrezia Zerillo, Concetta Claudia Coletta, Jessica Raffaella Madera, Gabriella Grasso, Angelapia Tutela, Pasquale Vito , Romania Stilo*, Tiziana Zotti*

* Corresponding authors: RS romstilo@unisannio.it; TZ tzotti@unisannio.it

This document includes:

Supplementary Figure 1

Supplementary Figure 2

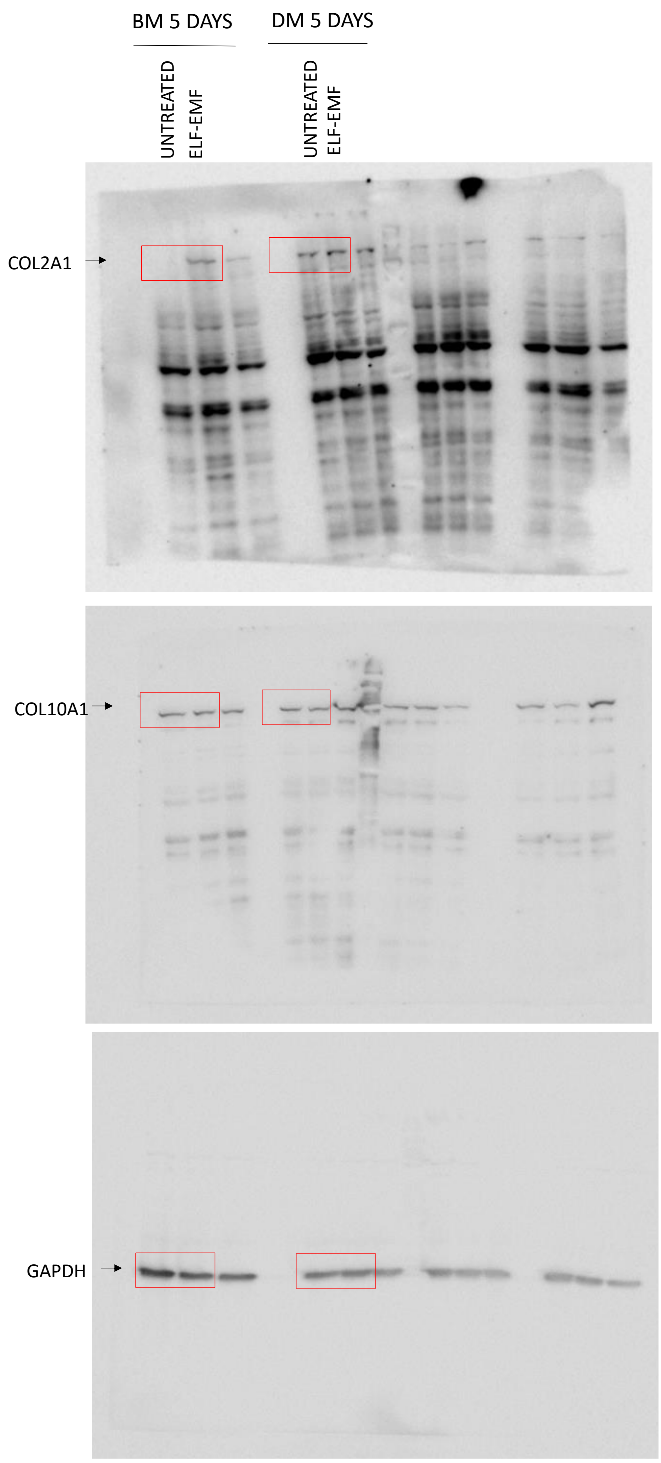

Supplementary Figure 1.

Uncropped full-length gels corresponding to Figure 2B

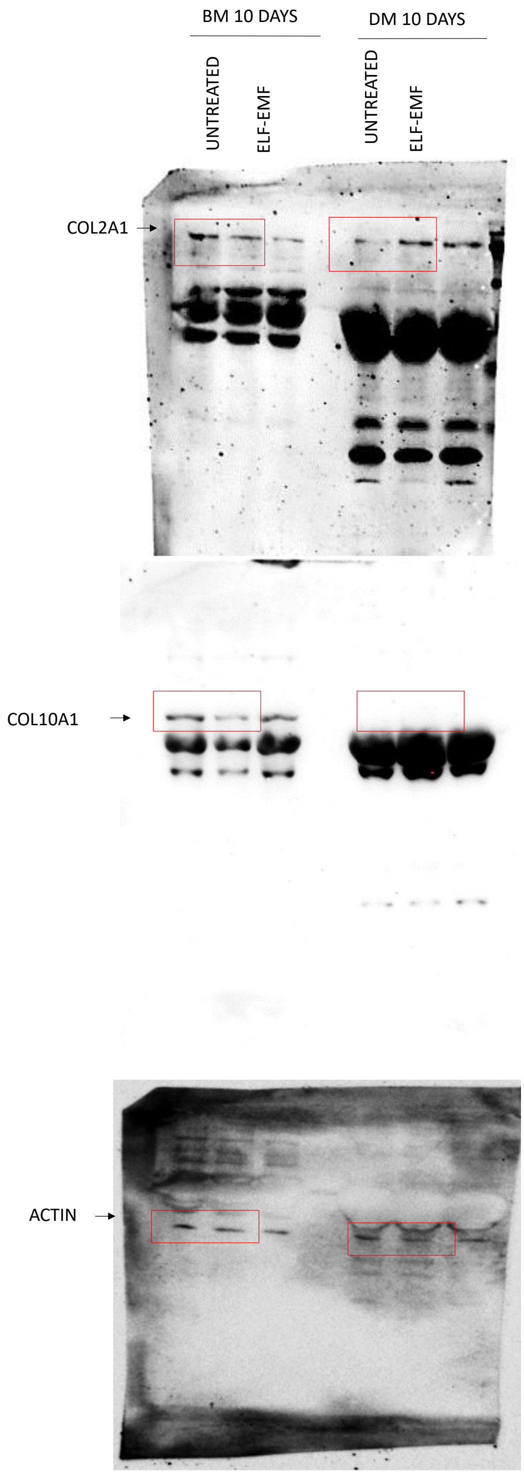

Supplementary Figure 2.

Uncropped full-length gels corresponding to Figure 4C
